# Electronic Health Record–Oriented Knowledge Graph System for Collaborative Clinical Decision Support Using Multicenter Fragmented Medical Data: Design and Application Study

**DOI:** 10.2196/54263

**Published:** 2024-07-05

**Authors:** Yong Shang, Yu Tian, Kewei Lyu, Tianshu Zhou, Ping Zhang, Jianghua Chen, Jingsong Li

**Affiliations:** 1 Research Center for Data Hub and Security Zhejiang Laboratory Hangzhou China; 2 Engineering Research Center of EMR and Intelligent Expert System, Ministry of Education College of Biomedical Engineering and Instrument Science, Zhejiang University Hangzhou China; 3 Key Laboratory for Biomedical Engineering of Ministry of Education College of Biomedical Engineering and Instrument Science, Zhejiang University Hangzhou China; 4 Kidney Disease Center The First Affiliated Hospital, Zhejiang University School of Medicine Hangzhou China

**Keywords:** knowledge graph, electronic health record, ontology, data fragmentation, data privacy, knowledge graphs, visualization, ontologies, data science, privacy, security, collaborative, collaboration, kidney, CKD, nephrology, EHR, health record, hypernym, encryption, encrypt, encrypted, decision support, semantic, vocabulary, blockchain

## Abstract

**Background:**

The medical knowledge graph provides explainable decision support, helping clinicians with prompt diagnosis and treatment suggestions. However, in real-world clinical practice, patients visit different hospitals seeking various medical services, resulting in fragmented patient data across hospitals. With data security issues, data fragmentation limits the application of knowledge graphs because single-hospital data cannot provide complete evidence for generating precise decision support and comprehensive explanations. It is important to study new methods for knowledge graph systems to integrate into multicenter, information-sensitive medical environments, using fragmented patient records for decision support while maintaining data privacy and security.

**Objective:**

This study aims to propose an electronic health record (EHR)–oriented knowledge graph system for collaborative reasoning with multicenter fragmented patient medical data, all the while preserving data privacy.

**Methods:**

The study introduced an EHR knowledge graph framework and a novel collaborative reasoning process for utilizing multicenter fragmented information. The system was deployed in each hospital and used a unified semantic structure and Observational Medical Outcomes Partnership (OMOP) vocabulary to standardize the local EHR data set. The system transforms local EHR data into semantic formats and performs semantic reasoning to generate intermediate reasoning findings. The generated intermediate findings used hypernym concepts to isolate original medical data. The intermediate findings and hash-encrypted patient identities were synchronized through a blockchain network. The multicenter intermediate findings were collaborated for final reasoning and clinical decision support without gathering original EHR data.

**Results:**

The system underwent evaluation through an application study involving the utilization of multicenter fragmented EHR data to alert non-nephrology clinicians about overlooked patients with chronic kidney disease (CKD). The study covered 1185 patients in nonnephrology departments from 3 hospitals. The patients visited at least two of the hospitals. Of these, 124 patients were identified as meeting CKD diagnosis criteria through collaborative reasoning using multicenter EHR data, whereas the data from individual hospitals alone could not facilitate the identification of CKD in these patients. The assessment by clinicians indicated that 78/91 (86%) patients were CKD positive.

**Conclusions:**

The proposed system was able to effectively utilize multicenter fragmented EHR data for clinical application. The application study showed the clinical benefits of the system with prompt and comprehensive decision support.

## Introduction

The fragmentation of patient data across multiple hospitals adversely impacts health care quality. In practice, patients visit different hospitals for various medical services. Previous studies have indicated that up to 26.5% of patients from a hospital have visited other institutions in the past 12 months [1-3]. These visits result in fragmented patient data across different hospitals within various electronic health record (EHR) systems [2,4-6]. Because of the sensitivity of medical data, sharing information between hospitals encounters obstacles related to data privacy and security [7]. As a result, EHR data in each hospital are often incomplete, making collaboration difficult.

The missing information from outside the local institution cannot provide clinicians with complete clinical evidence during routine practice. This potentially affects decision-making and harms health care quality in several aspects [2,8-10]:

- Delayed or missed diagnoses: Missing evidence from other hospitals can lead to unconsidered diseases until apparent symptoms occur.
- Duplicate care or additional tests: Additional tests may be ordered to verify diagnoses, even though records in other hospitals might already contain the needed results.
- Incomprehensive analysis and decisions: Because of incomplete disease history, clinicians may neglect important risk factors during decision-making.

The missing information could adversely affect patients in nearly 50% of cases. Much of the needed data can be found outside local hospitals [9]. Thus, utilizing multicenter fragmented EHR data for comprehensive decision support is essential while maintaining data privacy.

The knowledge graph stands as an explainable artificial intelligence method applicable across numerous domains. Using knowledge graphs to enhance semantic relationships within EHR data and execute deductive reasoning aids in producing understandable results within clinical practice [11]. Recent research on EHR-based knowledge graphs highlights the benefits of integrating medical knowledge into clinical applications (Textbox 1).

**Benefits of integrating medical knowledge into clinical applications.**

**1. Generating medical knowledge graphs from electronic health record data**

- Li et al [12] have introduced systematic methodologies for the semiautomatic construction of medical knowledge graphs using electronic health record (EHR) data. Entity recognition and occurrence-based algorithms play pivotal roles in relation to extraction and ranking.
- Hong et al [13] have introduced a clinical knowledge extraction technique using sparse embedding regression with multicenter EHR data. The embedding vectors derived from multicenter EHR data enhance the robustness of knowledge and facilitate the identification of data set heterogeneity.

**2. Knowledge graph–based EHR query**

- Thukral et al [14] have pioneered a method to convert tabular format EHR data into a knowledge graph representation, thereby enriching the semantic relationships among EHR data elements. This approach enables the execution of complex data queries using the easily interpretable SPARQL language.
- Xiao et al [15] used Ontology-Based Data Access to establish a virtual fast health care interoperability resources–based knowledge graph derived from Observational Medical Outcomes Partnership (OMOP) EHR data. This approach facilitates data interoperability with exceptional efficiency and generality.
- Although these studies greatly enhance data interoperability, they do not incorporate decision support functions.

**3. EHR knowledge graph–based clinical decision support**

- Carvalho et al [16] integrated EHR data with knowledge graph embeddings to develop a machine learning model for predicting intensive care unit readmissions. The knowledge embedded within EHR data serves as a feature for model training, resulting in improved predictive performance.
- Liu et al [17] introduced a heterogeneous similarity graph neural network approach for diagnosing predictions based on graph-formatted EHR data. The heterogeneous EHR graph undergoes normalization into multiple homogeneous graphs, which are then fused into a graph neural network to enhance prediction accuracy.

Although the methods described in Textbox 1 have shown enhanced performance, the model lacks both explainability and generality. In our previous studies, we introduced an EHR-oriented knowledge graph system, leveraging medical information often overlooked or underutilized by clinicians. This system aimed to offer decision support for diseases spanning multiple departments [18]. Specifically, it aided nonnephrology clinicians in identifying patients at risk of chronic kidney disease (CKD) who had been overlooked for extended periods. By transcending traditional knowledge barriers, the system tapped into previously underutilized information to facilitate the early detection of diseases crossing departmental boundaries.

Our previous work focused solely on cross-departmental data within a single hospital. However, in real-world scenarios, fragmented patient records within a single hospital often fail to provide sufficient information for knowledge graphs to conduct comprehensive analyses [10,19,20]. This limitation can result in imprecise decisions or delayed diagnoses, thereby constraining the practical implementation of knowledge graphs. Currently, only a handful of studies on multicenter knowledge graphs address the challenge of data fragmentation during model application phases. The predominant focus of multicenter knowledge graph research lies in 3 key areas: constructing knowledge graphs from diverse sources, completing knowledge graphs using data from multiple centers, and facilitating data interoperability guided by knowledge graphs [21-24]. Challenges in multicenter knowledge graphs encompass data heterogeneity, knowledge inconsistency, and concerns regarding the privacy and security of data sources. To address these challenges, researchers have explored federated knowledge graph embedding methods, allowing model training with multicenter data while upholding data security. For instance, Chen et al [25] introduced FedE, a knowledge graph embedding method leveraging a federated learning framework. In these approaches, each data source learns embedding vectors using its local data and then shares these vectors for model iteration. Peng et al [26] introduced FKGE (Federated Knowledge Graphs Embedding), which enables the learning of embeddings from various knowledge graphs in an asynchronous and peer-to-peer manner while safeguarding privacy. These methods have demonstrated improved performance in link prediction tasks without necessitating the centralization of original data. The studies, however, used multicenter data solely during the training phase of the embedding model and did not address data fragmentation during application. When applied to real-world decision support scenarios, these models are still fed with fragmented patient data from single hospitals only, which can significantly impact the performance of otherwise well-trained models. Therefore, it is crucial to empower knowledge graph systems to leverage multicenter fragmented EHR data for CDS while ensuring the preservation of data privacy, particularly in chronic disease management and long-term decision support applications.

Collaborative research networks such as the Observational Health Data Sciences and Informatics (OHDSI) offer valuable insights into addressing this issue [27,28]. These networks use local analysis results from various institutions, aggregating models, or summarizations to enhance generalizability and mitigate bias. Collaborative research does not necessarily require the centralization of original data, thus ensuring data security. Axfors et al [29] and Baigent et al [30] showcased meta-analyses by incorporating results from multiple randomized control trials and providing additional insights through summarization. Noman et al [31] and Tian et al [32] introduced collaborative methods using federated learning and multivariate aggregation to enhance model accuracy and generalizability. Such collaborative research only necessitates the collection of local analysis results and has demonstrated significant clinical value through the utilization of multicenter data sources.

Taking inspiration from collaborative research, this study introduces an EHR-oriented knowledge graph system designed to effectively harness multicenter fragmented patient EHR data while safeguarding data security. Implemented within each hospital, the system conducts local reasoning based on local EHR data and generates intermediate reasoning results. Through a distribution module, the intermediate results are formulated as an online subgraph with encrypted identities, facilitating multicenter collaboration and alignment. A blockchain network synchronizes the findings across centers, and the collaborated patient clinical evidence is used for final reasoning, enabling comprehensive CDS. Importantly, the original data remain within the local institute to uphold data security. The main contribution of the study is as follows:

- Introducing a novel framework for multicenter collaborative reasoning using fragmented EHR data for comprehensive CDS without the need to share original data. This approach enables knowledge graphs to use intact evidence for CDS purposes.
- Implementing an EHR-oriented knowledge graph system across multiple hospitals to standardize local EHR data and facilitate the local reasoning process. This initiative establishes a standardized semantic environment conducive to multicenter collaborative reasoning.
- Developing a distribution component and online subgraph structure to facilitate the collaboration of intermediate reasoning findings across multiple centers. This initiative addresses data privacy concerns and enhances local systems with the capability for multicenter collaboration.

An application study was conducted to evaluate the system’s effectiveness in assisting clinicians in detecting undiagnosed CKD in patients who visited multiple hospitals. The system successfully issued timely CKD warnings, a capability not supported by data from a single hospital alone.

## Methods

### EHR-Oriented Knowledge Graph System for Multicenter Collaboration

#### Overall System Architecture

**Figure 1:**
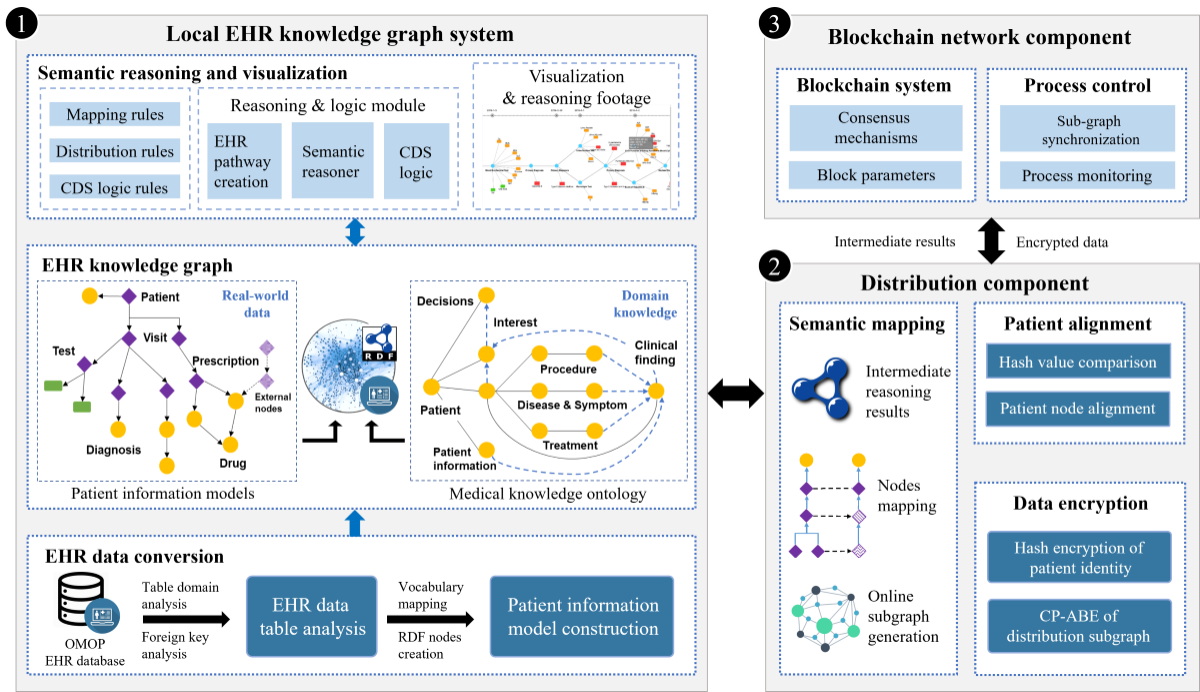
The architecture of the EHR-oriented knowledge graph system. (1) The local EHR knowledge graph system performs local reasoning based on local EHR data. (2) The distribution component creates online subgraphs with intermediate reasoning findings to synchronize across hospitals without sharing original EHR data. (3) The blockchain network supports the collaborative process. CDS: clinical decision support; CP-ABE: ciphertext-policy attribute–based encryption. EHR: electronic health record; OMOP: Observational Medical Outcomes Partnership; RDF: resource description framework.

This study presents an EHR-oriented knowledge graph system designed for multicenter collaboration using fragmented patient information. The overall system architecture is depicted in Figure 1. The proposed system uses structured EHR data following the Observational Medical Outcomes Partnership (OMOP) common data model (CDM) for semantic reasoning and clinical applications [33]. The semantic organization of EHR data within the knowledge graph adheres to the structure outlined in the OMOP CDM. The system consists of 3 main components: (1) the local EHR knowledge graph component, which conducts semantic reasoning on local EHR data to generate independent clinical findings; (2) the distribution component, which manages the distribution of intermediate reasoning results and patient alignment for multicenter collaboration; and (3) the blockchain component, which establishes a secure network for multicenter synchronization.

The system is deployed in hospitals, where it conducts local reasoning on local EHR data. The distribution component and blockchain network collaborate on intermediate reasoning results without exposing original data for privacy concerns. Subsequently, fragmented patient information from multiple hospitals is used to generate comprehensive CDS with complete clinical evidence.

#### The Local EHR Knowledge Graph Component

The local EHR knowledge graph component offers the capability to leverage local EHR data for semantic reasoning and CDS generation. This component has been adapted from our previous study [18]. The EHR Data Conversion Module is responsible for transforming EHR data into resource description framework (RDF)–type triples to enable semantic querying and reasoning. The module conducts an analysis of the EHR database and aligns table concepts with the entities within the knowledge graph ontology. Within the EHR knowledge graph, EHR data and clinical knowledge entities undergo a semantic transformation, organized under a unified top-level ontology structure. Semantic triples are used within the EHR knowledge graph to represent the clinical information pertaining to each patient. The Semantic Reasoning Module offers rule-based reasoning capabilities on the local knowledge graph to generate CDS-related findings. Additionally, the module establishes an EHR pathway for each patient to facilitate the collaboration of multicenter information. This involves connecting intermediate findings from multiple centers along a virtual timeline using semantic relationships, ultimately contributing to the final reasoning process for CDS. The Visualization and Explanation Module furnishes clinicians with a visualized timeline, aiding in their comprehension of critical medical information and evidence pertinent to the CDS.

To support the collaboration of local reasoning results, the generated findings are transformed into hypernym concept expressions to isolate original EHR data (eg, using “abnormal blood potassium” to represent reasoning findings from hyperkalemia diagnosis, blood potassium test results, or treatment medicines). Other hospitals will learn about the abnormality but not the original examination data or prescriptions. During the construction of the knowledge graph, candidate hypernym concepts are selected based on hierarchical relationships and the cosine similarity of their leaf nodes. Clinical experts review and adjust these candidate hypernym concepts to ensure information accuracy. The identified findings are automatically transformed into hypernym expressions during the knowledge graph reasoning process. Additional technical details are in Multimedia Appendix 1.

#### The Distribution Component

**Figure 2:**
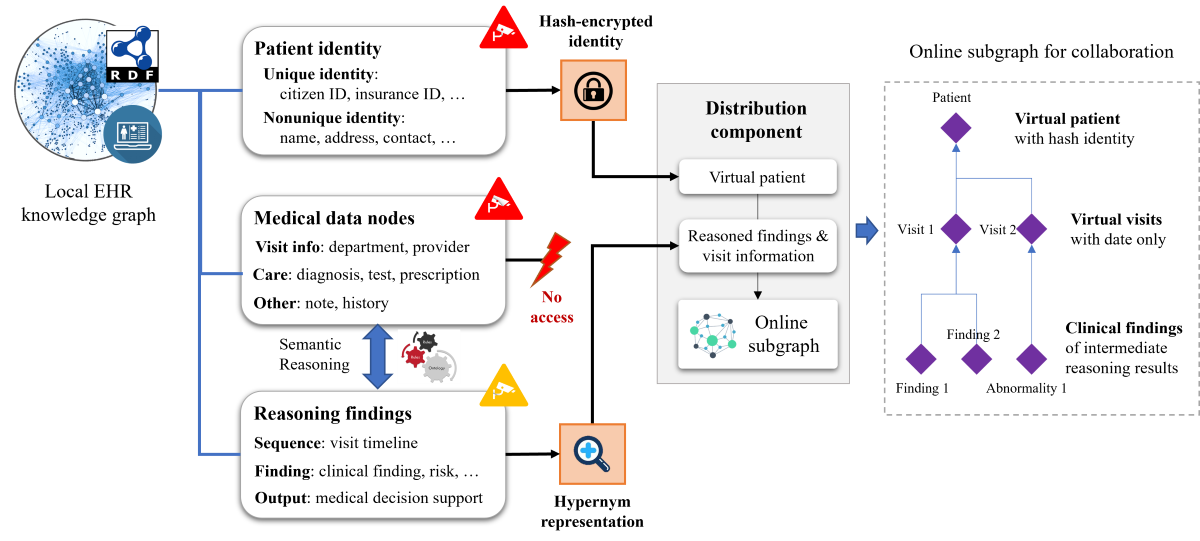
Data access of the Distribution Component. The original EHR data and identities are not acquired for online subgraph creation. The encrypted identity, visit time stamps, and authorized findings are permitted to be constructed as online subgraphs for collaborative reasoning. EHR: electronic health record.

The distribution component facilitates collaborative reasoning between multicenter EHR knowledge graph systems. It extracts intermediate reasoning results and visit pathway information, encrypts patient identities, and builds an online subgraph to synchronize local patient findings with other institutions. As shown in Figure 2, patient identities are hash encrypted for multicenter patient alignment. The hash codes of patient identities are compared to align the same patient across different hospitals, allowing multicenter findings for the same patient to be matched. Medical data nodes are prohibited from being distributed and are not used for online subgraph construction. Intermediate reasoning findings, represented by hypernym concepts, are extracted to build the online subgraph. This allows the transfer of a patient’s clinical evidence without exposing original EHR data. Other hospitals receive the online subgraph to collaborate on multicenter clinical evidence by loading intermediate findings of the aligned patients. If access control is required, the online subgraph can be encrypted using ciphertext policy attribute–based encryption.

#### The Blockchain Network Component

The blockchain network component establishes a blockchain node and manages the blockchain network. It securely synchronizes locally generated online subgraphs with other systems and delivers acquired triples from other hospitals to the local system, supporting the collaborative reasoning process. A requirement of the collaborative reasoning process is broadcast through the blockchain network, allowing each node to receive the process’s series number. The blockchain method was chosen because it is a proven approach for synchronizing data in distributed systems and is already used in medical domain studies [34,35]. All actions on the blockchain are logged and traceable. In this study, we used Golang [36] and libp2p [37] for blockchain platform construction, with proof of stake as the consensus mechanism.

### Multicenter Collaboration Settings of the EHR-Oriented Knowledge Graph System

#### Deployment Overview

**Figure 3:**
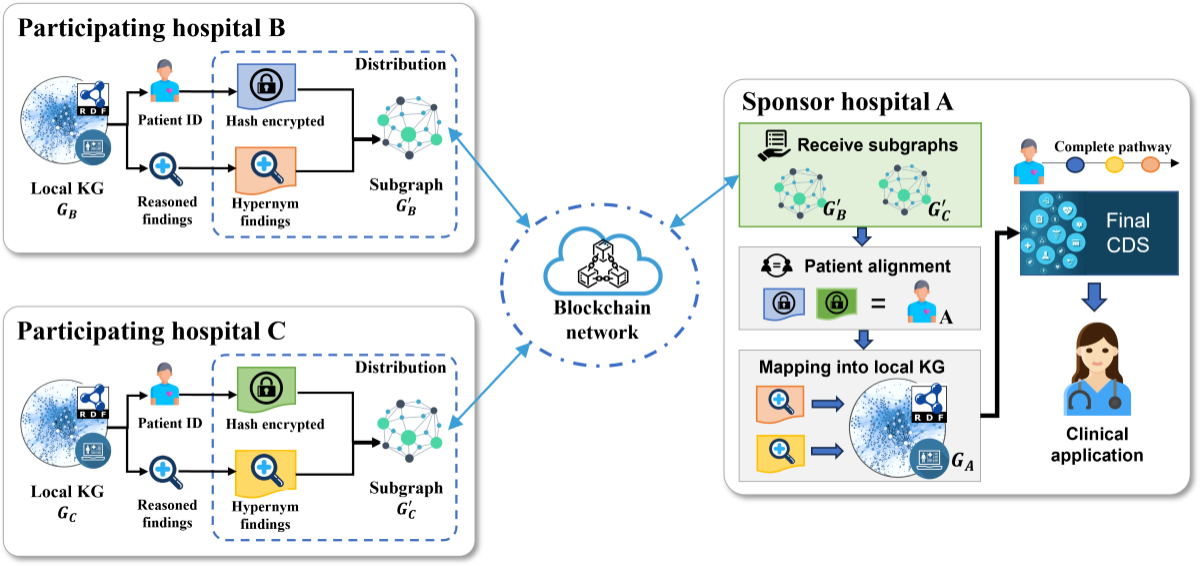
Setting of the EHR knowledge graph system in the multicenter environment. The participating hospitals perform local reasoning and pass the intermediate reasoning results through generated subgraphs. The sponsoring hospital performs local reasoning based on local EHR data and acquired intermediate findings to generate a comprehensive CDS for application. CDS: clinical decision support; EHR: electronic health record; KG: knowledge graph.

The EHR knowledge graph systems are deployed in each hospital and connected through a blockchain network for collaborative reasoning. Figure 3 illustrates the multicenter collaboration setting of the system. The EHR knowledge graph system is implemented in local hospitals and uses local EHR data sets for reasoning without exposing the original EHR data. Participating hospitals generate intermediate reasoning results, represented by hypernym concepts to isolate them from the original data, and use hash-encrypted identities to build online subgraphs. The sponsoring hospital receives the online subgraphs via the blockchain network and conducts patient alignment by comparing identity hash codes. For every patient, a comprehensive clinical pathway is established by amalgamating local evidence and intermediate reasoning outcomes from participating hospitals. A conclusive summary reasoning process utilizes the entirety of patient data to furnish clinicians with comprehensive CDS. Throughout this process, the original EHR data remain preserved within the local systems.

#### Patient Information Model

**Figure 4:**
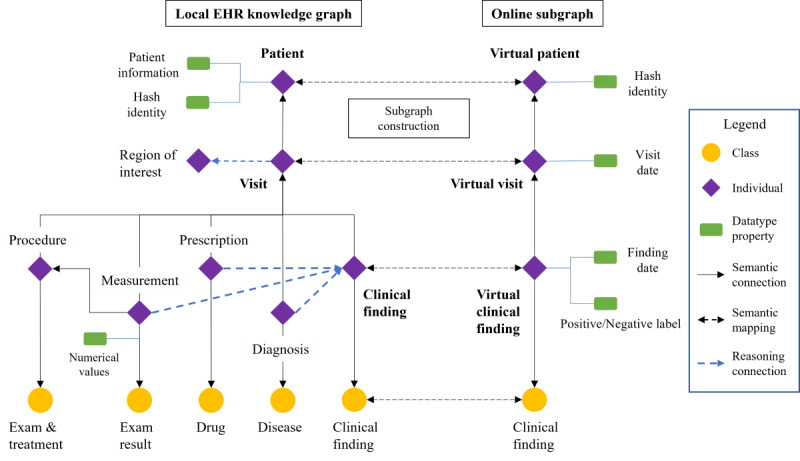
The semantic structure of the RDF-type patient EHR data. In the local EHR knowledge graph, a patient-visit-treatment structure defines the semantic structure of EHR information. In the online subgraph, the structure contains only patient nodes with hash identity, virtual visit nodes with visit dates, and virtual finding nodes with finding types and positive labels. EHR: electronic health record; RDF: resource description framework.

The EHR data within an OMOP CDM–based data table undergo transformation into RDF-type triples, thereby adopting a patient-centric information model suitable for querying and semantic reasoning. The structure of this patient information model is depicted on the left side of Figure 4. It is a 3-level, patient-visit-treatment semantic structure. It models each patient’s EHR data into a semantic clinical trajectory, facilitating patient-level querying and reasoning. The patient information model transmutes table-based EHR data into semantic graphs, enabling semantic reasoning, with each data element linking to its corresponding knowledge nodes.

#### The Online Subgraph

The online subgraph serves as a streamlined patient information model designed for online synchronization among multiple EHR knowledge graph systems. The ontology structure of the online subgraph is illustrated in the right section of Figure 4. The entities within the online subgraph mirror those within the local EHR knowledge graph, focusing solely on information pertinent to collaborative reasoning to conserve network resources. Each patient entity comprises solely hash-encrypted identity values for patient alignment. Virtual visit nodes exclusively feature visit dates to denote visit records from other hospitals. Similarly, virtual clinical finding nodes harbor intermediate reasoning outcomes tailored for collaborative reasoning purposes.

### The Multicenter Collaborative Reasoning Process

#### Purpose

**Figure 5:**
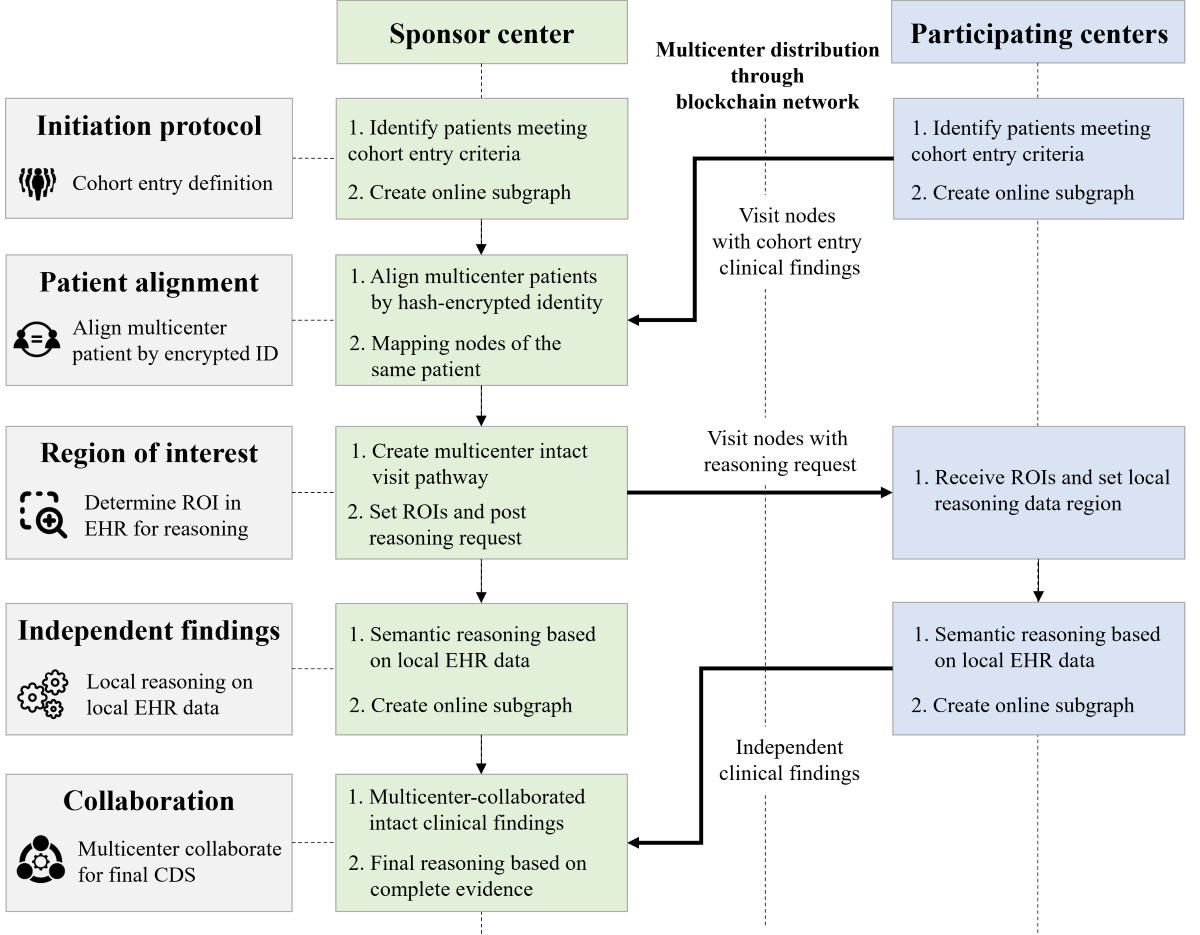
The overall process of collaborative reasoning. (1) All the centers identify patients meeting the cohort entry criteria. (2) The sponsoring center aligns patients by hash-encrypted identities. (3) The system creates a complete visit pathway with cohort entry findings for each patient to determine the ROIs for further reasoning. (4) Each center performs semantic reasoning and generates intermediate findings based on local EHR data. (5) The intermediate reasoning findings from multiple hospitals are collaborated for final decisions. CDS: clinical decision support; EHR: electronic health record; ROI: region of interest.

The multicenter collaborative reasoning process delineates a systematic interaction protocol for multicenter systems to engage in collaborative CDS reasoning. Illustrated in Figure 5, the process encompasses multiple steps, including preparing the reasoning cohort, aligning patients, defining the reasoning data period, and conducting semantic reasoning. This procedural framework ensures the efficacy and efficiency of collaborative reasoning endeavors.

#### Initiation Protocol

To initiate a collaborative reasoning process, the local system first executes the initiation protocol as a preparatory step. This protocol is tailored for the local system to delineate the reasoning cohort. The process identifies initial clinical evidence to determine whether a patient needs to join the collaborative reasoning. Unrelated patients are ruled out to save resources. The initiation protocol varies from one disease to another.

For example, in an application concerning unconsidered CKD warnings based on multicenter EHR data, the initiation protocol mandates each local system to ascertain whether a patient possesses kidney function test results but has not undergone nephrology visits. If this criterion is met, the patient becomes eligible to participate in the collaborative reasoning process. Subsequently, the local system generates virtual visit nodes containing abnormal kidney function findings for synchronization and collaborative analysis.

#### Patient Alignment

The patient alignment process is facilitated by the distribution component of the system. Patients are aligned by comparing hash-encrypted identities between local patient nodes and the received online subgraphs. Sensitive patient identities undergo hash encryption, ensuring that matching can be accomplished solely using ciphertext. This approach prevents the leakage of privacy information during data transmission and alignment. The distribution component maintains a table that records the mappings between matched patient node uniform resource identifiers. The records within the table serve as the foundation for mapping intermediate findings in subsequent steps. Subsequently, the reasoning outcomes pertaining to the matched patient are integrated into the local EHR knowledge graph system to facilitate collaborative reasoning.

#### Region of Interest Designation

The region of interest (ROI) delineates the disease-related observation period within the patient’s EHR data, specifying a temporal window for collaborative reasoning. The sponsoring hospital consolidates the results of the initiation protocol reasoning to identify observation periods relevant to disease risks. Collaborative reasoning concentrates on EHR data within this designated period to ensure efficient analysis and excludes irrelevant noise information.

The sponsoring hospital initially obtains visits and clinical findings related to the initiation protocol from other hospitals. Subsequently, the local EHR knowledge graph system constructs a multicenter visit timeline incorporating significant clinical findings. Following this, the system engages in semantic reasoning to ascertain the periods during which the patient’s data are pertinent to the disease and require additional collaborative reasoning for CDS. The ROIs are delineated and transmitted to other hospitals. Any nonrelated periods are subsequently excluded to optimize efficiency and conserve computing resources.

#### Local Reasoning Process

Throughout the collaborative reasoning process, the local reasoning process assumes responsibility for leveraging the local EHR data within the local knowledge graph. Its primary task involves generating atomic, independent clinical findings conducive to synchronization. The generated clinical findings exclusively present conclusions derived from EHR data. Intermediate findings use higher-level concepts to extract reasoning results from the original data, with the selection of these concepts being determined by domain experts and medical professionals. Other hospitals solely receive intermediate reasoned conclusions and are not provided with the corresponding original data or informed about the methodology used to derive the findings. For instance, the identification of abnormal blood potassium might be reasoned based on hyperkalemia, measurement results, or treatment medications, yet the other hospitals remain unaware of the specific source behind the reasoned findings.

The Reasoning Module conducts rule-based semantic reasoning, analyzing diagnoses, medical test results, procedures, and prescriptions separately at each visit to produce independent clinical findings. These findings are then converted into hypernym representation, aligning with semantic relationships within the knowledge graph, to facilitate multicenter collaboration. For instance, a <abnormal kidney function> node might represent a <estimated glomerular filtration rate at g3b stage> node within the knowledge graph.

#### The Multicenter Distribution and Summarization

**Figure 6:**
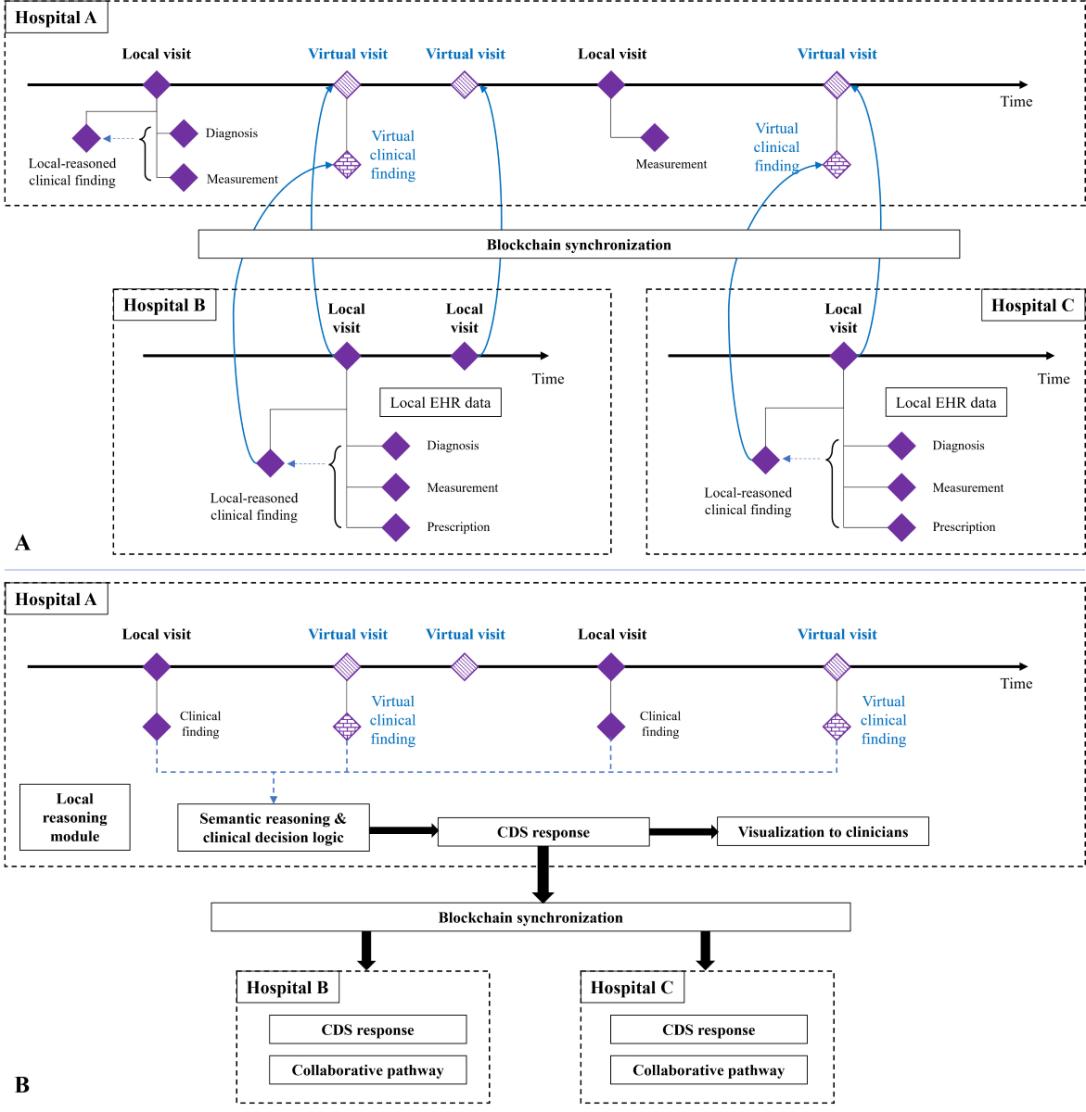
(A) Local systems generate intermediate reasoning results and collaborate by a blockchain network. (B) The multicenter collaborated findings support CDS reasoning and provide explainable results to clinicians. CDS: clinical decision support; EHR: electronic health record.

The sponsoring hospital conducts summarization reasoning, drawing upon intact clinical evidence to generate final CDS responses. An illustration outlining the distribution and summarization process is provided in Figure 6.

The online subgraphs transmit intermediate reasoning results to the sponsoring hospital for collaborative result synthesis. Upon receiving the subgraphs, the sponsoring hospital maps the incoming visits and clinical findings as virtual visits and virtual clinical findings, respectively. This process culminates in the creation of an integrated medical pathway for each patient, all achieved without the necessity of gathering original medical data. The system engages in semantic reasoning using multicenter collaborated reasoning outcomes. Collaborating on intermediate findings furnishes comprehensive patient clinical evidence, empowering the knowledge graph to produce precise decision support. The resulting CDS responses are presented in a timeline format, accompanied by explanatory reasoning details, allowing clinicians to review and interpret the information effectively.

In instances where decision support necessitates evidence beyond the scope of semantic reasoning, the system offers an interface to interact with other nonreasoning protocols to obtain the requisite evidence. For instance, in the application study, the garbled circuit algorithm is used to compare 2 test results without revealing the actual numerical values [38]. The protocol incorporates its own security mechanism to generate clinical findings in a data-private manner.

### Application to Unconsidered CKD Detection Through Fragmented EHR Data

#### Background

An application study assesses the performance and clinical value of the proposed system. The EHR knowledge graph system conducts collaborative reasoning on patients’ fragmented EHR data to identify their CKD-related risks, which were challenging to recognize using data from individual hospitals alone.

CKD is a prevalent chronic disorder associated with various complications and has seen a significant increase in prevalence in recent decades [39]. Epidemiological research indicates that the prevalence of CKD in China stands at 10.8%, yet only 12.5% of impacted individuals are aware of their condition [40]. The early detection of CKD relies on nonnephrology clinicians; however, it is particularly challenging because early-stage CKD often exhibits fewer symptoms. However, insufficient CKD knowledge among nonnephrology clinicians may result in the oversight of CKD-related risks during routine practice. Moreover, the extended monitoring required for the chronic progression of abnormal kidney function presents challenges for clinicians in timely identifying CKD [41,42]. Patients frequently seek care at multiple hospitals or clinics over a period, resulting in fragmented renal function test results and a disjointed disease history spread across different institutions. For nonnephrology clinicians with access solely to single-center data, identifying overlooked CKD becomes challenging. This situation can lead to delayed diagnosis, the necessity for repeated tests, and potentially inappropriate treatment. Combining fragmented test results and clinical findings while ensuring data security can facilitate the timely identification of CKD [43].

#### Application Study Design

In the application study, our system was deployed across 3 tertiary A-level hospitals in Hangzhou: the First Affiliated Hospital, College of Medicine, Zhejiang University (FAHZU); Zhejiang Hospital; and the Affiliated Hospital of Hangzhou Normal University (AHHNU). FAHZU is a comprehensive hospital providing a wide range of general care services. Zhejiang Hospital and AHHNU specialize in providing focused care services. By combining these hospitals, the focus is on addressing the needs of patients who seek care at multiple health care facilities for various types of medical services.

**Figure 7:**
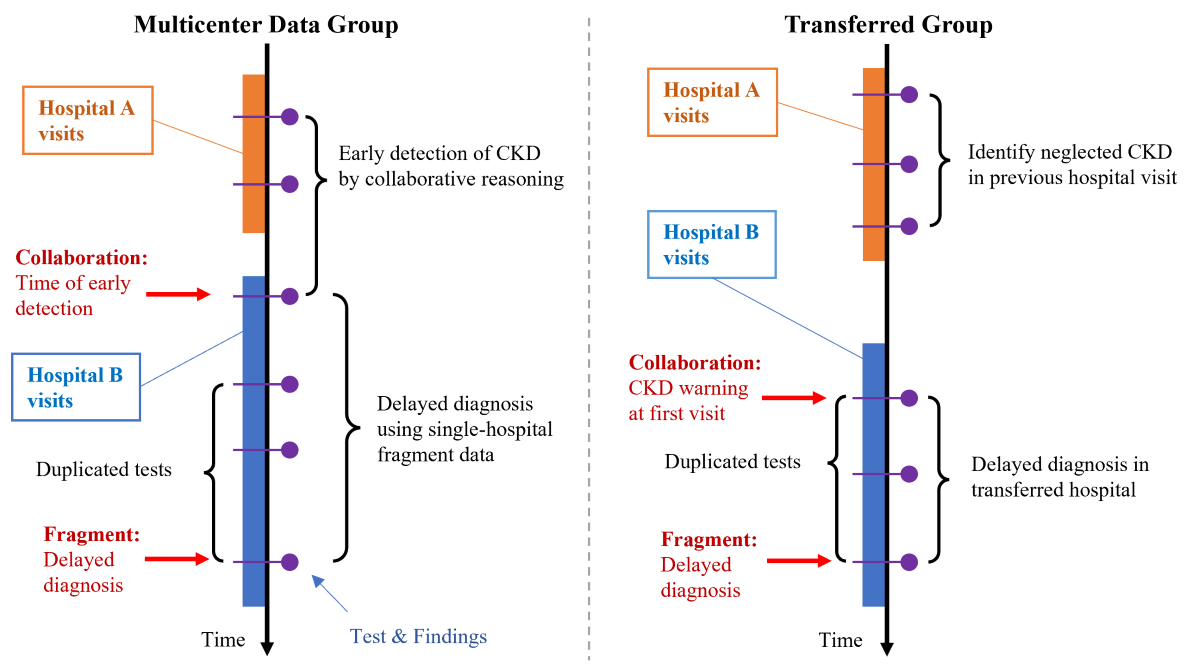
The multicenter data group focuses on the early detection of unconsidered CKD using collaborated evidence from multiple hospitals. The transferred group focuses on unconsidered CKD warning at the first visit in the transferred hospital by collaborating evidence from previous hospital visits. CKD: chronic kidney disease.

The study conducted collaborative reasoning on fragmented medical information from patients who had visited multiple hospitals, aiming to detect overlooked CKD without necessitating the gathering of original EHR data. The study concentrated on patients in nonnephrology departments and leveraged CKD-related information typically overlooked by nonnephrology clinicians. This approach aimed to facilitate early detection of CKD risks. As illustrated in Figure 7, the collaborative reasoning mainly focused on 2 types of patients meeting the CKD diagnosis criteria (Textbox 2).

A disease-specific local ontology for CKD and semantic reasoning rules were developed based on clinical practice guidelines and CKD management studies [44-47]. Medical experts from the kidney department of FAHZU contributed to the creation of the ontology and semantic rules to ensure accuracy and clinical functionality. The systems analyzed local test results related to kidney function and identified abnormal findings, along with occurrences of visits. Primary CKD–related evidence is detailed in Multimedia Appendix 2.

**Focus of the collaborative reasoning.**

**1. Detection through the combination of information from multiple hospitals**

These patients’ chronic kidney disease (CKD)–related data are dispersed across multiple hospitals, and only collaborative reasoning that integrates findings from multiple centers can promptly identify CKD. The collaboration of clinical findings could fulfill the 3-month monitoring criteria for chronic development. (This group is labeled the “Multicenter Data Group” in the “Results” section.)

**2. Detection by bridging the information gap between hospitals**

These patients fulfilled the CKD diagnosis criteria during previous visits to one hospital but were overlooked by clinicians. Subsequently, when transferred to another hospital without access to their prior data, health care providers neglected the patients’ CKD risks for an extended period. (This group is labeled the “Transferred Group” in the “Results” section.)

#### Evaluation and Data Source

The evaluation of the application study encompasses the following aspects of clinical value and system performance:

First, the system’s capacity to identify overlooked patients with CKD using multicenter EHR data within a secure environment is assessed. Nephrology experts evaluate the reasoning outcomes by examining the comprehensive multicenter EHR data to determine whether the patients have been confirmed as CKD positive. The assessment involved identifying patients who had subsequent EHR data regarding kidney function after the date recorded in the EHR when the knowledge graph identified the patients as meeting the CKD criteria. In essence, the assessment process utilizes EHR data both from the knowledge graph used for reasoning and subsequent EHR data used as “labels.” As the study cohort lacks CKD diagnosis labels, it is crucial to incorporate additional data beyond the utilization of the knowledge graph to ensure the patients truly exhibit CKD positivity “in the future.” Relying solely on identical data for assessment merely evaluates whether the system adheres to guideline-based rules and generates appropriate outputs. The selected subset, augmented with additional data, facilitates a more comprehensive assessment and supports the subsequent evaluation aspects.

Second, the advantages offered by collaborative reasoning in terms of discovery lead time, risk coverage, and potential test reduction are evaluated. Discovery lead time highlights the system’s capability for early CKD detection, suggesting its potential to mitigate delayed diagnoses. The lead time *t*_lead(M)_ of the multicenter data group is computed as the difference between the date *t*_CDS_ of the visit where the knowledge graph system identified patients meeting the CKD diagnosis criteria and the date *t*_diagnosis_ of the assessment when clinicians diagnosed CKD using single-hospital EHR data for these patients, as in the following equation:

*t*_lead(M)_=*t*_diagnosis_ – *t*_CDS_ **(1)**

For patients in the transferred group, the lead time *t*_lead(_*_T_*_)_ is calculated as the difference between the date *t*_transfer_ of the first visit to the transferred hospital and the date *t*_diagnosis_ of the assessment when clinicians diagnose CKD using EHR data from the transferred hospital, as in the following equation:

*t*_lead(T)_=*t*_diagnosis_ – *t*_transfer_ **(2)**

The risk coverage demonstrates the system’s capability to furnish abundant evidence of CKD for explanation and review by clinicians. Collaborative reasoning yields CKD-related risks *r* from multiple-hospital EHR data within a 3-month window at the ROI (*t*_ROI_). As a baseline, single-hospital reasoning provides risks using EHR data from the latest 3 months (*t*_3_*_m_*) as a comparative baseline. The risk coverage *cov* is calculated as a comparison as follows:



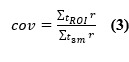



The duplicate examination reduction demonstrates the potential of the system to decrease unnecessary renal function tests for CKD diagnosis through multicenter collaborative reasoning. It is calculated based on the additional test records used in the clinician assessment of single-hospital data. The duplicated tests are depicted in Figure 7.

Lastly, the visualization and explanation of decision support were emphasized. A user interface was developed for information review and explanation of CDS results. An example of an overlooked patient with CKD identified through collaborative reasoning was presented to demonstrate the functionality of the visualization and explanation. The evaluation of blockchain performance is detailed in Multimedia Appendix 3 to underscore its suitability for the collaborative reasoning process.

The application study used EHR data from March 2008 to November 2020, provided by FAHZU, Zhejiang Hospital, and AHHNU. The study cohort comprised patients who had visited at least two of these hospitals. Cohort patients were selected and aligned using hash-encrypted identities. Patients with at least one decreased kidney function test result were included in the cohort. Patients who had either a nephrology visit or a kidney disease diagnosis record during the observation period were excluded. The EHR data, initially in the OMOP CDM format, were converted into the local EHR knowledge graph system.

### Ethics Approval

The study was approved by the Clinical Research Ethics Committee of FAHZU (approval number 2020-330) and was exempt from informed consent for the following reasons: (1) the identity information of the data was either removed or encrypted before utilization; (2) the study did not involve commercial interests, and the data were not publicly disclosed; and (3) the data were used solely for system evaluation, and the study did not impact the health status of the patients.

## Results

### Study Cohort Characteristics

The cohort for multicenter reasoning of unconsidered patients with CKD included individuals from FAHZU, Zhejiang Hospital, and AHHNU, spanning from March 2008 to November 2020. All patients had visit records at FAHZU as well as visit records at either Zhejiang Hospital or AHHNU. Patients with a test record of an estimated glomerular filtration rate lower than 60 mL/min or a urine albumin-to-creatinine ratio higher than 30 mg/g were included in the cohort. Exclusion criteria were having a diagnosis record of any kidney disease or having a visit record from the kidney department. The cohort comprised a total of 1185 patients. Table 1 presents the characteristics of the patients at the time of cohort entry.

**Table 1:** Characteristics of the study cohort.

Characteristics		Values	
**Target cohort**			
	Patients, n	1185	
**Age (years)**			
	18-59, n (%)	219 (18.48)	
	>60, n (%)	966 (81.52)	
	Mean (SD)	68.52 (11.35)	
**Sex, n (%)**			
	Female	470 (39.66)	
	Male	715 (60.34)	
**Measurement, mean (SD)**			
	Blood potassium (mmol/L)	4.27 (0.46)	
	Serum creatinine (μmol/L)	109.24 (59.86)	
	Estimated glomerular filtration rate (mL/min)	57.20 (16.81)	
	Blood urine nitrogen (mmol/L)	7.55 (3.64)	
	Blood glucose (mmol/L)	5.97 (1.92)	
	High-density lipoprotein cholesterol (mmol/L)	1.21 (0.37)	
	Low-density lipoprotein cholesterol (mmol/L)	2.53 (0.92)	
	Total cholesterol (mmol/L)	4.60 (1.13)	
	Albumin-to-creatinine ratio (mg/mmol)	44.69 (110.36)	
**Diagnosis, n (%)**			
	Diabetes mellitus	160 (13.50)	
	Hypertension	281 (23.71)	
	Cardiovascular disease	106 (8.95)	
	Hyperlipidemia	29 (2.45)	
**Visit department^a^ (top 10 most), n (%)**			
	Cardiovascular medicine	130 (11.43)	
	Emergency department	119 (10.47)	
	Gastroenterology	64 (5.63)	
	Ophthalmology	59 (5.19)	
	Orthopedics	56 (4.93)	
	Endocrinology	56 (4.93)	
	Urology	54 (4.75)	
	Respiratory medicine	50 (4.40)	
	Cardiology	46 (4.05)	
	Infectious disease	39 (3.43)	

^a^The percentages were calculated based on the 1137 patients who had a specific visit department at the cohort entry visit. In total, the cohort included visits to 39 different departments.

### Multicenter Reasoning of Unconsidered CKD

The evaluation study results are presented in Table 2. The EHR knowledge graph systems performed collaborative reasoning across the 3 hospitals and identified 124 patients who met the CKD diagnosis criteria based on the combination of multicenter medical information. In the multicenter data group, 69 patients met the CKD diagnosis criteria through the collaborative reasoning of fragmented EHR data. The data from either hospital alone would not support the diagnostic criteria for CKD. In the transferred group, 55 patients met the CKD diagnostic criteria during early visits to one hospital, but their CKD positivity was identified much later at another hospital. Clinicians overlooked their CKD risks during the initial hospital visits. The information gap prevents doctors at subsequent hospitals from making prompt diagnoses. Collaborative reasoning could alert clinicians to previously neglected CKD risks during the initial visit.

A total of 91 patients were assessed by nephrology clinicians. These patients were selected if subsequent EHR data were available beyond what were used by the knowledge graph reasoning. These additional EHR data served as “labels” to further confirm CKD. The results demonstrated that the proposed system effectively identified patients’ CKD risks through collaborative reasoning. The false positives in the assessment were primarily due to CKD recovery. These patients had undergone surgery or long-term treatment, resulting in temporarily reduced kidney function. After the treatment ceased and no longer affected the renal system, kidney function recovered, leading to false positives. Nonetheless, these patients required kidney function monitoring at the time of reasoning to prevent chronic risks.

**Table 2:** Evaluation results of collaborative reasoning of patients with unconsidered CKD^a^.

Group	Patients, n	Assessed patients^b^, n	Confirmed CKD by assessment, n (%)	Unconfirmed CKD by assessment, n
Patients meeting CKD diagnosis criteria (full cohort)	124	91	78 (86)	13
Multicenter data group^c^	69	40	32 (80)	8
Transferred group^d^	55	51	46 (90)	5

^a^CKD: chronic kidney disease.

^b^The patients’ CKD was assessed by clinicians using subsequent electronic health record data that were dated later than the reasoned CKD date in accordance with CKD guidelines.

^c^The CKD status of patients in this group was determined through collaborative reasoning using findings from multiple hospitals.

^d^The CKD of patients in this group was overlooked at one hospital, and the information regarding their CKD risk did not transfer to another hospital during subsequent visits.

### Advantages of Multicenter Reasoning

Table 3 presents the benefits of collaborative reasoning compared with single-hospital data analysis, encompassing discovery lead time, risk coverage comparison, and duplicate examination reduction. The discovery lead time and potential examination reduction are calculated based on subsequent EHR data following the data used in the reasoning process. The discovery lead time demonstrated that the system could identify CKD risks early on, long before clinical assessment. By leveraging multicenter fragmented medical information, the system delivered timely CDSs for cross-departmental clinicians. Consequently, it has the potential to reduce delayed or missed diagnoses during routine practice and address information gaps stemming from data fragmentation and security issues.

**Figure 8:**
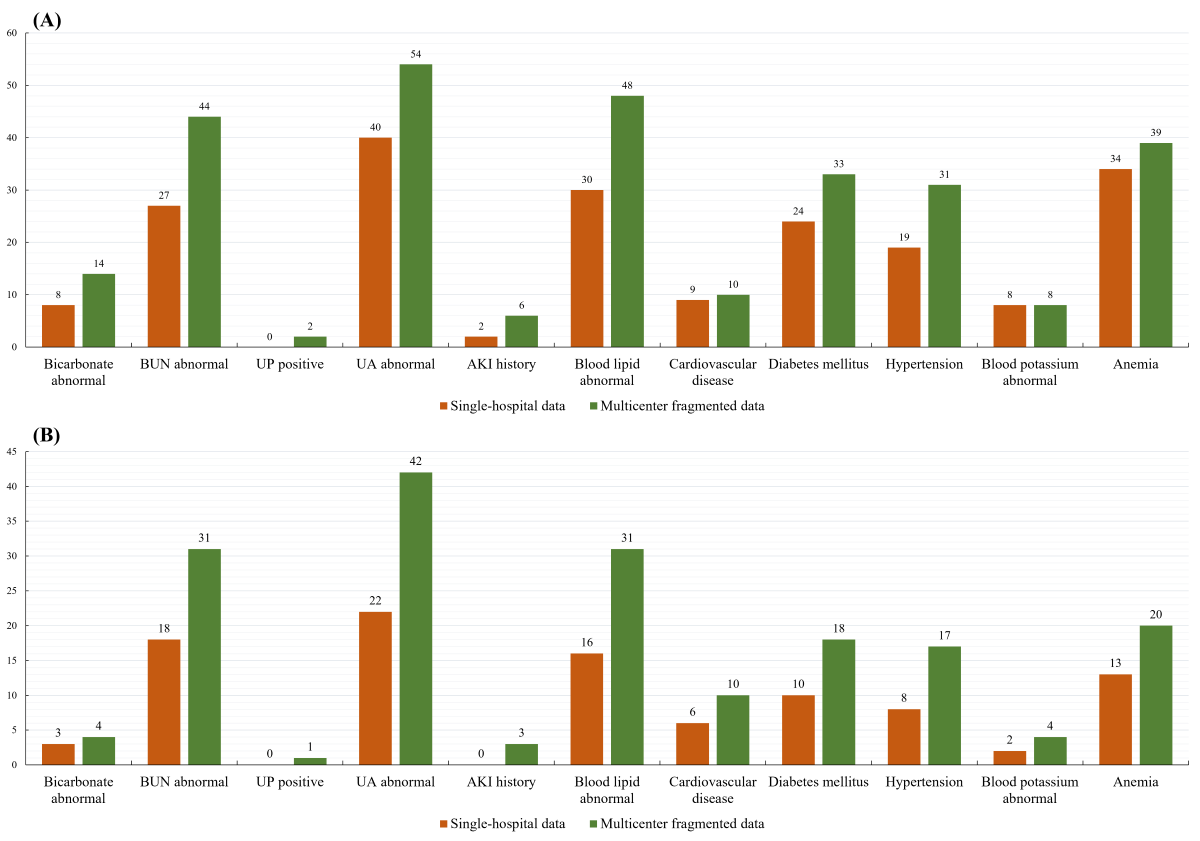
(A) Identified CKD-related risks of the Multicenter Data Group. (B) Identified CKD-related risks of the Transferred Group. The collaborative reasoning identified more CKD-related risk identification, while single-hospital reasoning missed some clinical evidence during decision support. AKI: acute kidney injury; BUN: blood urea nitrogen; CKD: chronic kidney disease; UA: uric acid; UP: urinary protein.

The CKD-related risk coverage highlights the system’s ability to furnish comprehensive information for clinicians to review and assess the significance and progression of CKD risks. Part of the comparison between identified CKD-related risks in multiple-hospital collaborative reasoning and single-center reasoning is depicted in Figure 8. Each column indicates the number of patients found to have the specific risk. For instance, a history of acute kidney injury (an essential risk factor for CKD) from other hospitals may go unnoticed in single-hospital reasoning, leading clinicians to miss a crucial reference point in evaluating the patient’s condition.

The potential examination reduction suggests that collaborative reasoning can effectively leverage multicenter fragmented information, using previous tests to identify overlooked CKD and offer decision support. This has the potential to reduce duplicate tests resulting from information gaps and facilitate prompt treatment.

**Table 3:** Discovery lead time and comparison between multicenter collaborative reasoning and single-center data analysis.

Variables		Multicenter data group	Transferred group
**Discovery lead time^a^ (days)**			
	Mean (SD)	434 (363)	208 (219)
	Median	364	121
**Reduced duplicate examinations^b^**			
	Tests, mean (SD)	3.34 (2.72)	3.56 (4.12)
**Risk coverage comparison^c^**			
	Ratio, %	133	165

^a^The discovery lead time of the multicenter data group is calculated as the difference between the date of chronic kidney disease reasoning finding and the date of clinician assessment results. Conversely, the discovery lead time of the transferred group was calculated as the difference between the date of the first visit to the transferred hospital and the date of clinician assessment results.

^b^Test reduction refers to the additional tests required for clinicians to assess chronic kidney disease based solely on single-hospital data, compared with using multicenter collaborative reasoning.

^c^Using single-center reasoned chronic kidney disease–related risks as the baseline.

### Visualization and Explanation of CDS Results

**Figure 9:**
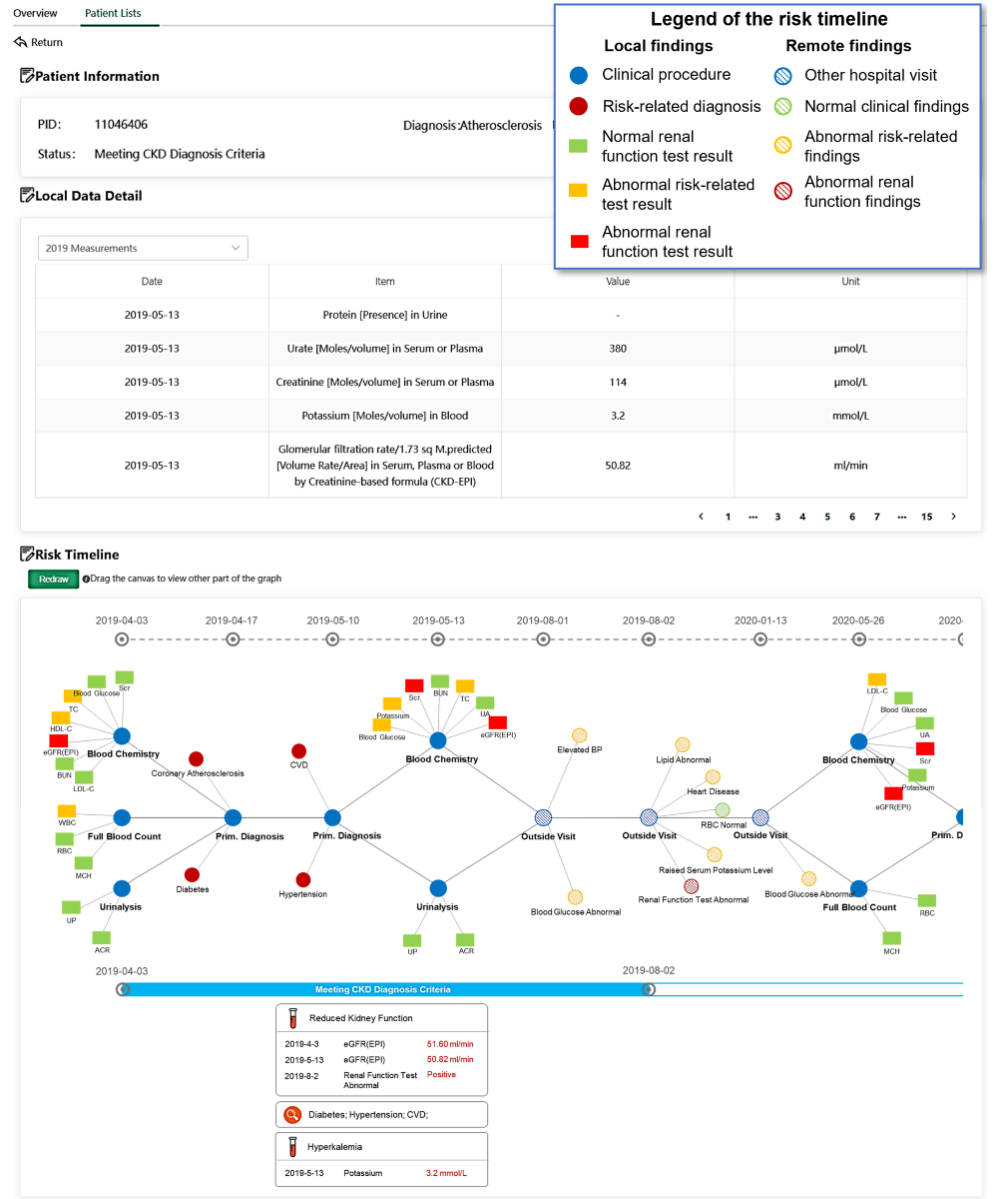
The system interface with information timeline and the reasoning footage for clinicians to review (translated version).

The user interface depicted in Figure 9 showcases an example of an overlooked patient with CKD identified by the system. At the top of the page, patient information and a table format of local EHR data are presented for review. On the lower part of the page, a graphical timeline illustrates the patient’s medical pathway, accompanied by reasoning footage providing an explanation of the CDS for overlooked CKD. Additional interfaces can be found in Multimedia Appendix 4.

The patient with ID 11046406 visited FAHZU in April 2019, May 2019, and May 2020, and visited other hospitals (Zhejiang Hospital in this case) in August 2019 and January 2020. The system established an ROI meeting the CKD diagnosis criteria from April 4, 2019, to August 2, 2020. Local findings and remote findings are distinguished by different filling styles. The local EHR data revealed abnormal estimated glomerular filtration rate test results and several CKD-related risks during that period. Findings from other hospitals indicated abnormal kidney function and several additional CKD-related risks. The system aggregated these findings and concluded that the patient met the CKD diagnosis criteria, exhibiting several significant risk factors. The reasoning footage and essential risks within the ROI are listed beneath the timeline for review. Despite the limited information provided by the remote system, it aids clinicians in identifying abnormalities and making targeted inquiries.

## Discussion

### Principal Findings

In this study, we introduced an EHR-oriented knowledge graph system designed for use in a medical information-sensitive environment. The proposed framework enables knowledge graph systems to collaborate on clinical evidence across multiple hospitals, facilitating comprehensive CDS without the need for model aggregation. Several studies have highlighted the significance of addressing data fragmentation in clinical decision-making for improving health care outcomes [19,48]. Our proposed pilot framework was designed to address this issue and was implemented in a real-world application scenario. The application demonstrated that the system could identify chronic disease risks using multicenter fragmented information, a capability that single-hospital reasoning alone cannot achieve.

As an enhancement to our previous study, we introduced a collaborative reasoning framework to the system. This framework adopts a decentralized design, eliminating the need for a coordination center and offering flexibility for implementation across hospitals [49]. The knowledge graph within the hospital is a fully functional system equipped with data transformation and semantic reasoning capabilities, enabling local reasoning and CDS. The distribution component and blockchain facilitate the participation of the local system in collaborative reasoning. The local knowledge graph is responsible solely for handling reasoning on local triples, while the distribution component manages the collaboration of the multicenter reasoning process.

The application study on warning unconsidered patients with CKD underscored the clinical value of the proposed system. Fragmented EHR data of a patient present challenges for clinicians in obtaining a comprehensive view of multicenter evidence, potentially resulting in delayed or missed diagnoses [8,9]. The proposed systems were capable of identifying overlooked CKD in advance, particularly benefiting nonnephrology clinicians who might overlook patients’ renal risks. The results of the application study indicated that the proposed system could (1) counter delayed or missed diagnoses, (2) potentially reduce redundant tests, and (3) provide complete information for clinicians to review.

During the application study, 69 patients were identified as meeting the CKD diagnostic criteria. Furthermore, clinician assessment revealed that 80% (32/40) of the evaluated patients exhibited positive CKD symptoms and test results. This suggests that a significant number of patients could benefit from the multicenter EHR knowledge graph system for timely CDS, thereby facilitating prompt treatment and enhancing health care quality. Conversely, another group of patients highlights the challenge posed by the information gap between hospitals. The system identified 55 patients with overlooked CKD during previous hospital visits. These patients either remained undiagnosed during subsequent visits to another hospital or received diagnoses much later. Patient transfers between hospitals often result in an information gap. The proposed system has the capability to transmit information between hospitals, alerting clinicians to risks and offering CDS during the initial visit after transfer.

The discovery lead time revealed that patients with overlooked CKD were neglected for an extended period. This underscores the detrimental effects of EHR data fragmentation across institutions. Valuable disease information remains obscured and underutilized due to fragmentation. Without comprehensive evidence, clinicians face limitations in identifying cross-departmental risks, leading to prolonged neglect. Implementing the proposed system across an extensive network encompassing medical centers and primary clinics has the potential to facilitate collaboration and early detection of disease risks by leveraging valuable information. Improved risk coverage can also furnish clinicians with a comprehensive background, enabling them to conduct thorough inquiries and assessments.

The study’s main concepts revolve around distributed local reasoning and the collaboration of intermediate reasoning results to facilitate comprehensive CDS. For an evidence-based approach, it is crucial to gather complete findings from multiple centers to elucidate the rationale behind decision support creation. However, information exchanges do occur during the collaborative process. The framework implemented 3 major data security measures: (1) It ensured isolation between reasoning findings and original data. The intermediate findings generated by the local reasoning process do not disclose the source of the data. Remote hospitals only receive analysis results (eg, abnormal blood potassium and abnormal blood glucose findings) without information about how these findings were derived, whether from diagnosis or measurement. (2) The clinical findings used for cross-hospital collaboration are high-level concepts carefully chosen by domain experts during the development of the disease’s local ontology. This selection aims to minimize the level of detail in the findings and obscure the relationship between reasoned findings and their original records. (3) The online subgraph is encrypted during the online synchronization phase, ensuring that only authorized hospitals on the network can receive the intermediate findings and protecting against cyberattacks. During clinical practice, clinicians also conduct inquiries to gather medical history from patients. Concerns about information exposure are manageable through patients’ authorization of medical record usage and the implementation of proper security measures.

While the application study concentrated on unconsidered CKD warning, the proposed system can be adapted to other application domains for various clinical purposes. For instance, leveraging multicenter information aids in the sensitive and precise identification of type 2 diabetes, while collaborative reasoning offers risk warnings for general practitioners, and so forth. We are committed to further enhancing the proposed system to ensure its reliability and security in real-world applications. Implementing a more implicit collaboration method would foster better system adoption, particularly in data-sensitive environments.

### Limitations

This study has its limitations. When deploying the system across an extensive network of hospitals and clinics, communication efficiency may encounter bottlenecks. Additionally, the network and computational resource costs may escalate due to patient alignment and semantic reasoning of numerous subgraphs. To address these challenges, further systematic design and application of the Hyperledger method could facilitate the widespread deployment of the system. Furthermore, the patient alignment process relies on unique identifiers, which may pose challenges when unique citizen IDs are absent in the records. To enhance system adoption, alternative approaches using nonunique identifiers for similar patient alignment are necessary [4].

### Comparison With Prior Work

In this study, we introduced a framework for knowledge graph systems to collaborate on multicenter fragmented clinical evidence to generate comprehensive CDS without sharing original data. First, our method focuses on collaborating local reasoning findings rather than original EHR data. By contrast, existing studies on patient record completion primarily concentrate on securely sharing EHR data through blockchain and selective encryption, encountering challenges related to data privacy and property rights [50,51]. Second, our proposed framework leverages multicenter fragmented information during the CDS application phase. Previous studies on using multicenter EHR data primarily focus on enlarging the model training set through federated learning to enhance model performance. However, these methods often fall short in addressing incomplete patient information from single centers when models are applied in daily practices [31,32,52]. Third, our proposed method uses knowledge graphs for explainable CDS and conducts local reasoning for local clinical findings. Current multicenter knowledge graph studies predominantly emphasize federated embedding learning, which trains embedding models without centralizing diverse knowledge graphs to ensure data security [25,26]. However, these methods also encounter challenges related to data incompleteness during model application.

To our knowledge, only a few studies have addressed the collaboration of fragmented medical information during CDS in practical settings. We introduced a pilot framework and reported clinical application results demonstrating the value of using multicenter fragmented information for CDS. This approach may assist nonnephrology clinicians in identifying patients with CKD risks in advance.

### Conclusions

This study introduced an EHR-oriented knowledge graph system for collaborative CDS. The research demonstrated that the system effectively leverages fragmented patient EHR data from multiple hospitals, enabling the generation of CDS with intact clinical evidence without the need to share original data, thus addressing security and privacy concerns. The application study showcased a valuable scenario of detecting overlooked CKD using multicenter clinical information. Patients derived benefits from collaborative CDS for early-stage chronic disease warnings, all while safeguarding data security, an aspect unsupported by single-hospital data.

## Appendix

Multimedia Appendix 1 Additional technical details of the proposed system.

Multimedia Appendix 2 The process of identifying patient with unconsidered CKD warning signs and primary primary evidence related to CKD. CKD: chronic kidney disease.

Multimedia Appendix 3 The performance and resource consumption of the blockchain network.

Multimedia Appendix 4 Additional user interfaces of the proposed system.

## Abbreviations

AHHNU: Affiliated Hospital of Hangzhou Normal University
CDM: common data model
CDS: clinical decision support
CKD: chronic kidney disease
EHR: electronic health record
FAHZU: First Affiliated Hospital, College of Medicine, Zhejiang University
FKGE: Federated Knowledge Graphs Embedding
OHDSI: Observational Health Data Sciences and Informatics
OMOP: Observational Medical Outcomes Partnership
RDF: resource description framework
ROI: region of interest

## Data Availability

The data used in the study are from and stored in 3 different hospitals. Because of the multicenter origin of the data, access to the data requires further approval from these hospitals and is not publicly available. Please contact the corresponding author to make reasonable requests on the data access.
