# Supplementary material for: Electronic Health Record–Oriented Knowledge Graph System for Collaborative Clinical Decision Support Using Multicenter Fragmented Medical Data: Design and Application Study
Source: J Med Internet Res. 2024 Jul 5;26:e54263. doi: 10.2196/54263 (PMC11259764; doi:10.2196/54263)
Supplement: Multimedia Appendix 1 [file jmir_v26i1e54263_app1.docx]

**Multimedia Appendix 1. Additional technical details of the development system.**

**1. The EHR knowledge graph**

The EHR knowledge graph system in each hospital contains a local EHR knowledge graph for storing RDF-type EHR data and clinical knowledge entities. The semantic representation of EHR data and relation to clinical knowledge support the semantic reasoning to generate intermediate findings.

The local knowledge graph consists of 2 parts: the patient information model $G_{p}$ with graph-expression of patient data, and the medical knowledge ontology $G_{m}$ with domain knowledge of medical concepts and relationships. The EHR knowledge graph in the local hospital is then $G=\left\{ G_{p},G_{m} \right\}$.

The patient information model, as illustrated in the article, is a semantic and structural representation of patient data. For each patient $p_{i}$, the clinical visits of the patient are constructed as entities $\mathcal{V}_{\boldsymbol{i}}=\left\{ v_{i}^{1},v_{i}^{2},\ldots,v_{i}^{t} | t_{j}<t_{j+1},j=1\ldots k \right\}$, where $v_{i}^{t}$ is one visit occurrence of the patient. Within each visit $v_{i}^{t}$, the clinical information is classified as disease history $\boldsymbol{D}_{\boldsymbol{h}}^{\boldsymbol{t-1}}$, chief complain $\boldsymbol{C}$, several treatment process $\boldsymbol{E}^{\boldsymbol{t}}$, and discharge summary $\boldsymbol{D}_{\boldsymbol{dis}}^{\boldsymbol{t}}$. The clinical processes in one visit are also created as entities with related information (e.g., diagnosed disease, measurement results, prescribed drugs, etc.) as

$$v_{i}^{t}=\left\{ \boldsymbol{D}_{\boldsymbol{h}}^{\boldsymbol{t-1}},\boldsymbol{C}^{\boldsymbol{t}},e_{1}^{t}\ldots,e_{m}^{t},\boldsymbol{D}_{\boldsymbol{dis}}^{\boldsymbol{t}} | \sum_{j=1}^{m} e_{j}^{t}=\boldsymbol{E}^{\boldsymbol{t}},d_{e_{j}}<d_{e_{j+1}} \right\}$$

where a single treatment process $e_{j}^{t}$ contains diagnosis $\left( D,D^{R} \right)$, measurement $\left( M \right)$, treatment $\left( Tre \right)$, and prognosis, $\left( M^{\left( p \right)},D^{\left( p \right)} \right)$, forming

$$e_{j}^{t}=\left\{ D,M,D^{R},Tre,M^{\left( p \right)},D^{\left( p \right)} \right\}$$

The EHR Data Conversion Module will follow the above semantic structure to transform local EHR data into semantic representation.

The medical knowledge ontology provides medical concepts and knowledge relationships for semantic reasoning and support the EHR data transformation. The medical knowledge ontology consists of several knowledge sources including SNOMED CT, ICD-10, LOINC, RxNorm, NCCD, Disease Ontology, SemMedDB, etc. The component defines a two-level ontology structure for medical knowledge. The top-level ontology defines the global structure for clinical knowledge and EHR data. The disease local ontology contains specific medical concepts, relationships, and rules for certain diseases and clinical purposes. The medical knowledge ontology provides standard clinical terms to support the knowledge graph system performs EHR data transformation in various hospitals and maintain knowledge consistency and data semantic consistency in multiples hospital. That is essential for the proposed framework to achieve collaborative reasoning using fragmented patient information in multiple hospitals.

**2. The multicenter collaboration of online subgraphs.**

The EHR knowledge graph system is implemented in the local hospital and connects to local EHR datasets for reasoning. Each online subgraph consists of 3 major parts: the hash encrypted patient identity $h\left( I_{p} \right)$, the virtual visit node $v^{'}\left( v_{d},c \right)$ containing basic visit information, and the intermediate reasoning findings $f^{'}$. The patient identity $I_{p}$ is hash encrypted by secure hash algorithm multicenter patient alignment, noted as $h\left( I_{p} \right)$. The hash code of patient identity is compared to align the same patient in different hospitals. The visit pathway contains virtual visit nodes $v^{'}\left( v_{d},c \right)$ consist of visit date $v_{d}$ and hospital identifier $c$. The intermediate reasoning findings $f^{'}$ represented by hypernym concepts are extracted to build the online subgraph, to transfer patient’s clinical evidence without exposing original EHR data. The online subgraph $G^{'}=\left\{ h\left( I_{p} \right),f^{'},v^{'}\left( v_{d},c \right) | I_{p},f^{'},v_{d},c\in G \right\}$ is a subset of local EHR knowledge graph $G$ using the same semantic structure and representation.

As in Figure 1, the sponsor hospital receives the online subgraphs through the blockchain network, and performs patient alignment by comparing identity hash codes. The online subgraphs are mapped into the local EHR knowledge graph as $G_{A}^{'}=G_{A}+G_{B}^{'}+G_{C}^{'}$. For each patient $p$, a complete clinical finding set $f_{p}=f_{a}+f_{b}^{'}+f_{c}^{'}$ is collaborated, where $f_{a}$ is the local reasoned findings in hospital A. A summary reasoning using $f_{p}$ is then performed for the final CDS with fragmented patient information from multiple hospitals.


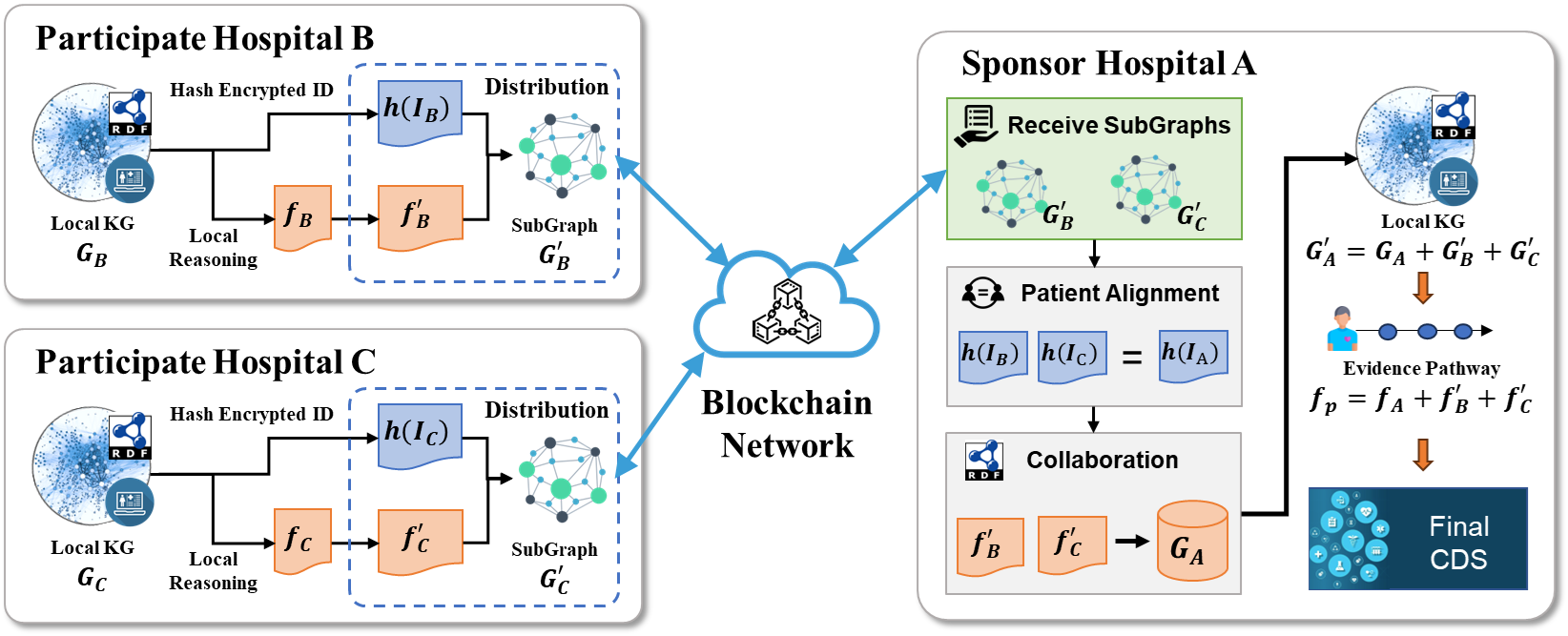


Figure 1. The collaboration of multicenter intermediate reasoning results through blockchain network and online subgraphs.
