# Supplementary material for: Electronic Health Record–Oriented Knowledge Graph System for Collaborative Clinical Decision Support Using Multicenter Fragmented Medical Data: Design and Application Study
Source: J Med Internet Res. 2024 Jul 5;26:e54263. doi: 10.2196/54263 (PMC11259764; doi:10.2196/54263)
Supplement: Multimedia Appendix 2 [file jmir_v26i1e54263_app2.docx]

**Multimedia Appendix 2. The process of the unconsidered CKD patient warning and primary CKD related evidence.**

An overall process is illustrated in Figure 1 and is described as follows: (1) Sponsor hospital will initialize a collaborative reasoning request and post the request through the blockchain network. (2) Sponsor hospital and participating hospitals start initiation protocols of unconsidered CKD patient warning. The local knowledge graph systems perform semantic reasoning on local EHR data and identify test results showing reduced kidney function. The local systems create online subgraphs containing encrypted patient identities, reduced kidney function findings and related visit occurrences. (3) The online subgraphs are synchronized to the sponsor hospital by the blockchain network. The patients are aligned based on encrypted identities. The sponsor hospital generates an overview of patients’ kidney function timeline and set ROIs according to chronic abnormalities or occasional abnormalities. The sponsor hospital then returns semantic reasoning requests of the visits within the ROIs back to participating hospitals by online subgraphs. (4) The sponsor hospital and the participating hospitals perform local reasoning of visits within the ROIs. The semantic reasoning modules generated CKD related clinical findings for each visit, including risk factors, consequences of kidney diseases and kidney function trends. Table 1 shows the CKD related findings used in the application study. The results are created as clinical findings and synchronized by online subgraphs. (5) The sponsor hospital gathers CKD related clinical findings by online subgraphs and performs final semantic reasoning for clinical decision support and visualization, giving the clinicians about the clinical significance of the unconsidered CKD patients.


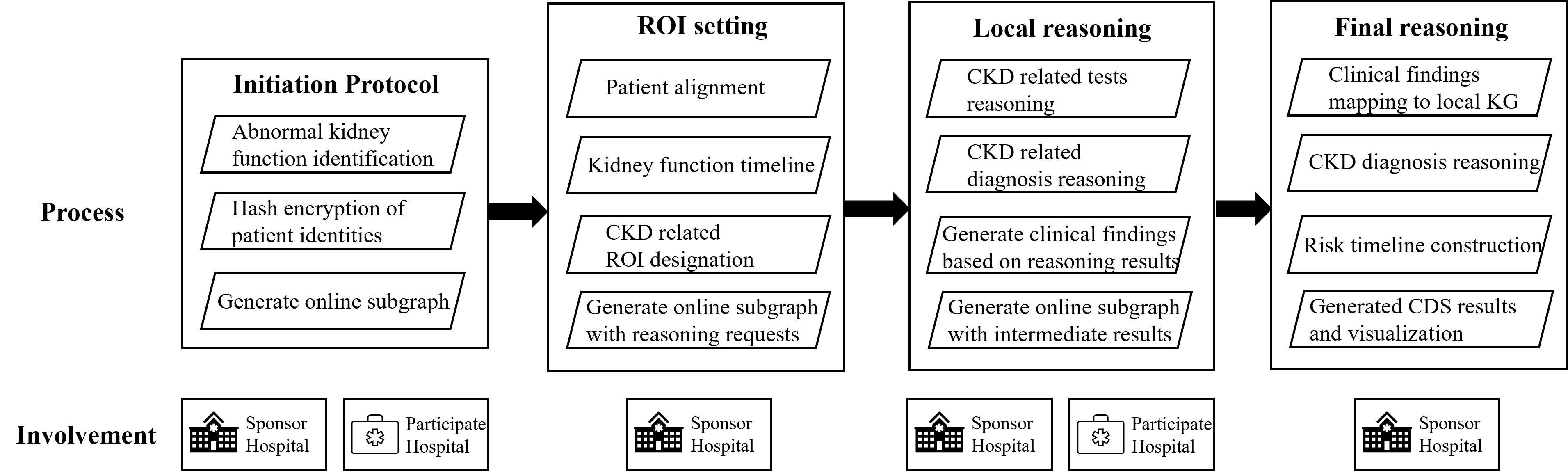


Figure 1. An overall process of the unconsidered CKD patient warning.

Table 1. CKD related clinical findings used in the application study.

| **Type** | **Characteristics** |
| --- | --- |
| Kidney function | Decreased kidney function trends ^a^ |
|  | Current kidney function lower than stage G3b ^b^ |
| Risk factors for CKD | Age over 60 years |
|  | Diabetes |
|  | Hypertension |
|  | Acute kidney injury history |
|  | Cardiovascular disease |
|  | Autoimmune disease |
|  | Cirrhosis |
|  | Abnormal urine acid |
|  | Abnormal urine protein |
|  | Abnormal blood lipid |
| Consequences of CKD ^c^ | Anemia |
|  | Abnormal bicarbonate |
|  | Abnormal parathyroid hormone |
|  | Hyperkalemia |
|  | Abnormal blood urine nitrogen |

^a^The trend is acquired by garbled circuit method, which can compare two numbers without knowing the value of the number.

^b^The stage is calculated based on local EHR data only. The sponsor hospital doesn’t acquire stage information from other institutes.

^c^The consequence of CKD may indicate a patient with missed diagnosis of CKD. Therefor the system considered these factors as clinical significance for clinicians to review.
