# Supplementary material for: Electronic Health Record–Oriented Knowledge Graph System for Collaborative Clinical Decision Support Using Multicenter Fragmented Medical Data: Design and Application Study
Source: J Med Internet Res. 2024 Jul 5;26:e54263. doi: 10.2196/54263 (PMC11259764; doi:10.2196/54263)
Supplement: Multimedia Appendix 3 [file jmir_v26i1e54263_app3.docx]

**Multimedia Appendix 3. The performance and resource consumption of the blockchain network.**

The statistics of the blockchain network performance during the collaborative reasoning process is shown in Table 1. The synchronization of online sub-graphs occurred between three hospitals. The total triples transferred on the network during the collaborative reasoning process are 356,750. The data volume, after encryption, is 52.50 MB in total. The time consumption is 17.87 seconds in total (including blockchain communication). The time consumption during synchronization was affordable for the multicenter reasoning of a patient.

Note that the performance of the blockchain network is not the focus of the study. The result is to show that the time consumption and network consumption of the blockchain synchronization is acceptable for the multicenter collaborative reasoning process.

Table 1. Blockchain network performance of the collaborative reasoning process.

| **Variables** | **Value** |
| --- | --- |
| Transferred triples during collaborative reasoning (n) | 356,750 |
| Data volume ^a^ (MB) | 52.50 |
| Time consumption |  |
| total consumption (seconds) | 17.87 |
| triples per second (n/sec) | 19963.63 |
| patients per second (n/sec) | 66.31 |

^a^The data volume was calculated after data encryption process.
