# Supplementary material for: Electronic Health Record–Oriented Knowledge Graph System for Collaborative Clinical Decision Support Using Multicenter Fragmented Medical Data: Design and Application Study
Source: J Med Internet Res. 2024 Jul 5;26:e54263. doi: 10.2196/54263 (PMC11259764; doi:10.2196/54263)
Supplement: Multimedia Appendix 4 [file jmir_v26i1e54263_app4.docx]

**Multimedia Appendix 4. Additional user interfaces of the system.**

The proposed system was already deployed in hospitals for demonstration. The system contains a control center for summarization of unconsidered CKD patients and the detailed clinical decision support interface shown in the article. Here we provide additional user interfaces of the system.

The Figure 1 shows the data overview of the system. It is a statistic summarization of the unconsidered CKD patients found by the system. Note that the summarization in the figure contains not only multicenter collaborative reasoning results but also single center findings from our previous study. The summarization shows the visit department distribution, primary diagnoses distribution and severity of the kidney function. The Figure 2 is the list of the unconsidered CKD patients. The users can search patients through patient ID, primary diagnoses, types of CKD risks (meeting CKD diagnostic criteria or at risk of CKD) and observation period. Through a hyperlink the clinicians can view the detail of the patient, which is the same interface of Figure 9 in the article.


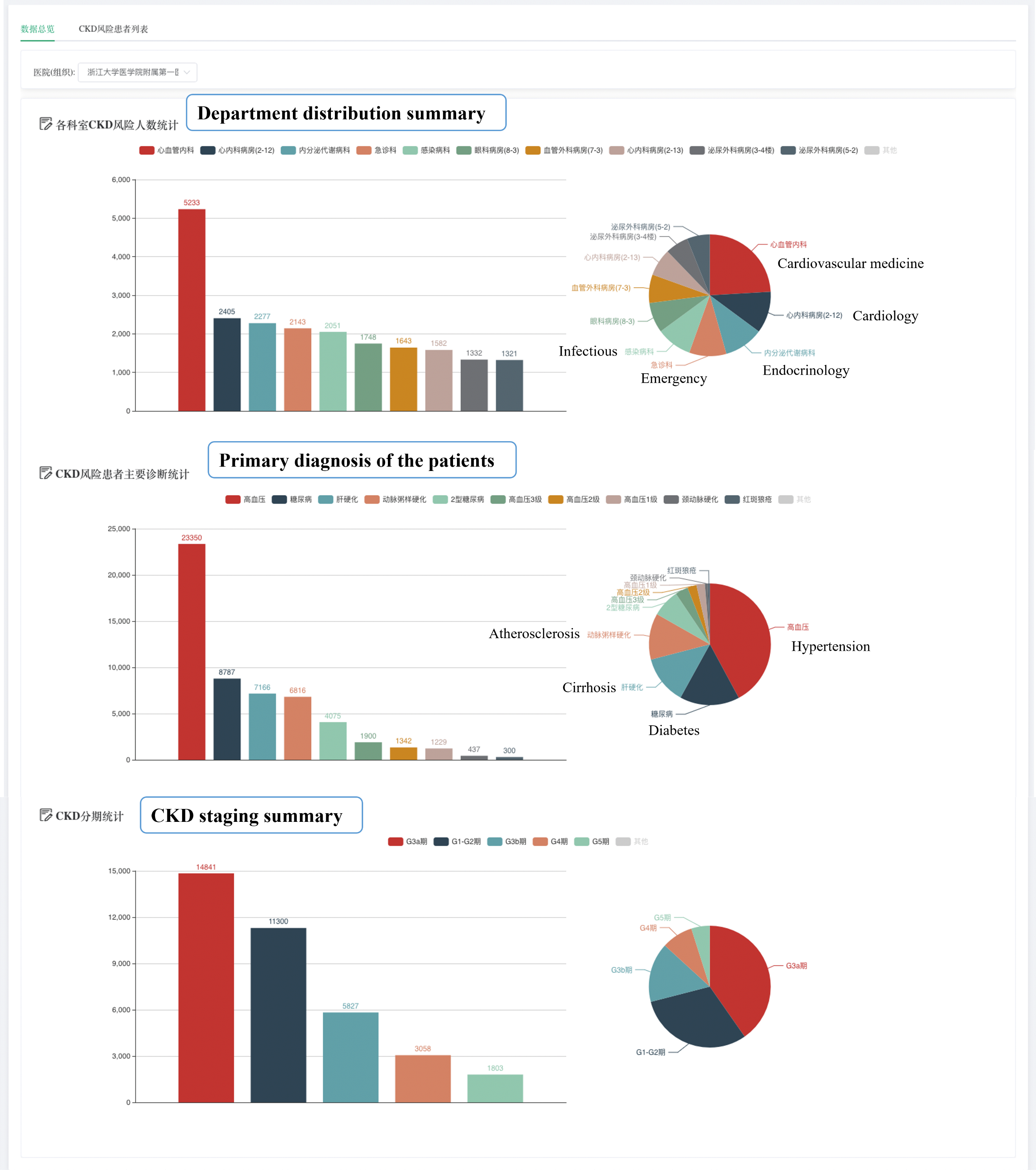


Figure 1. Statistic summarization interface.


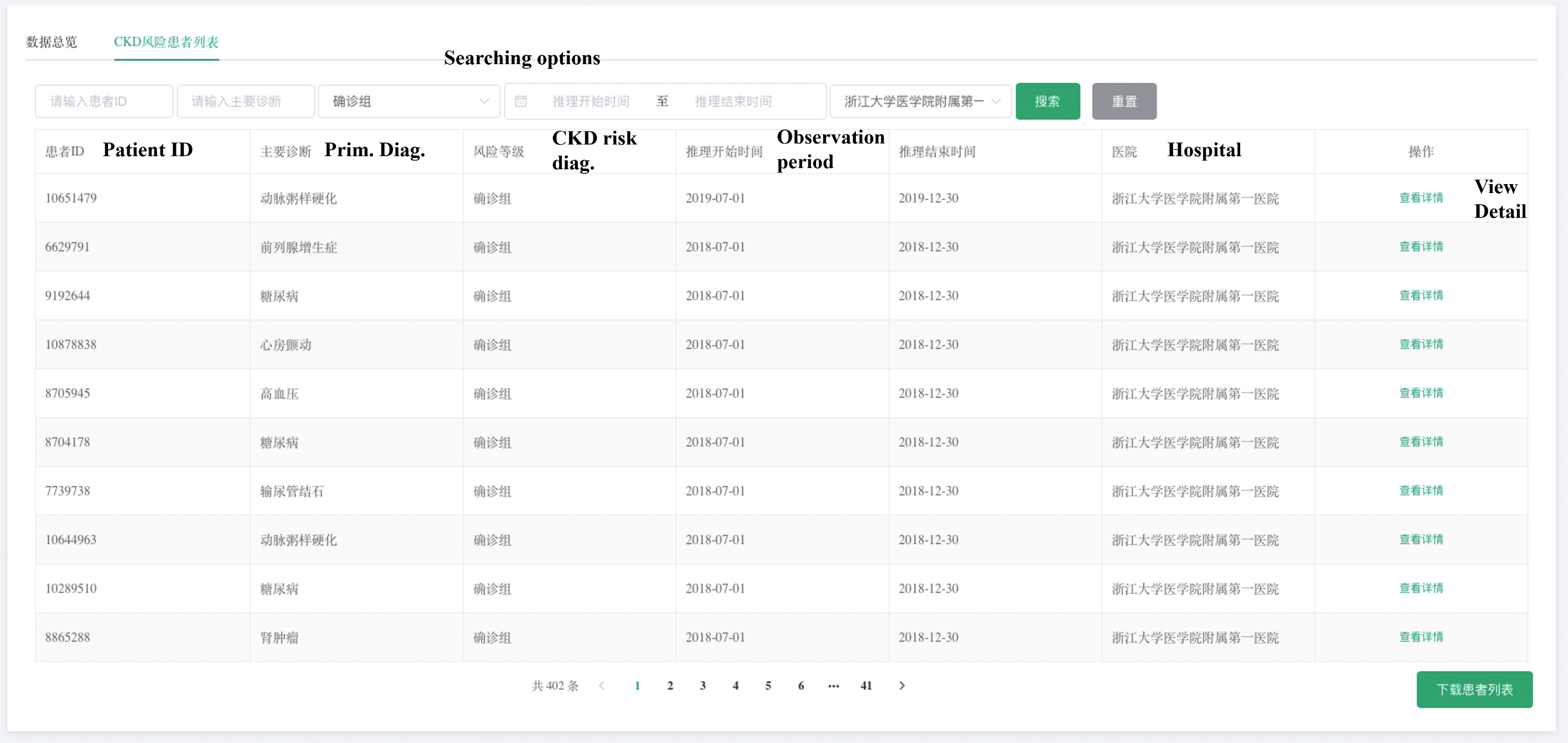


Figure 2. Patient list interface.
